# A Rare Case of Giant Gangrenous Lung Abscess

**DOI:** 10.7759/cureus.79090

**Published:** 2025-02-16

**Authors:** Georgi Yankov, Magdalena Alexieva, Zaharinka Makshutova, Radostina Cherneva

**Affiliations:** 1 Department of Thoracic Surgery, University Hospital “St. Ivan Rilski”, Medical University of Sofia, Sofia, BGR; 2 Department of Intensive Care, University Hospital “St. Ivan Rilski”, Medical University of Sofia, Sofia, BGR

**Keywords:** bilobectomy, conservative treatment, diagnostics, lung abscess, surgery

## Abstract

Gangrenous lung abscess (LA) is a limited form of lung gangrene. Although its frequency has decreased dramatically in recent years, it occupies an important place in clinical practice. Delaying its diagnosis or incorrect treatment leads to a risk to the patient's life. Timely diagnostics and treatment reduce morbidity and mortality rates. А 68-year-old woman who underwent a right upper bilobectomy for a gangrenous LA is presented. The case is considered in the context of the prolonged therapeutic course and the need for surgical treatment.

## Introduction

Acute infectious lung destructions are of three types: acute purulent abscess, extensive lung gangrene, and gangrenous abscess. Acute lung abscess (LA) occurs as a result of liquefactive necrosis of the lung parenchyma, leading to the formation of a cavity (larger than 2 cm) filled with necrotic debris or fluid, caused by bacterial infection [1]. Pulmonary gangrene, also known as necrotizing pneumonia, is a massive necrosis of the lung parenchyma, in which there is no tendency for clear demarcation. Gangrenous LA presents a limited form of pulmonary gangrene. In gangrenous abscesses, there is a greater likelihood of demarcation, but there is a tendency for the formation of abscess cavities. Clinical symptoms usually resemble pneumonia or are predominated by a systemic inflammatory response syndrome or a septic state. Multidrug-resistant microorganisms like *Klebsiella pneumoniae*, *Staphylococcus aureus*, *Streptococcus pneumoniae*, and *Pseudomonas aeruginosa*, etc., are some of the most important etiological factors for the development of the disease. The different resistance mechanisms contribute to the multiplication of polyresistant bacteria and the progression of pneumonia to LA, gangrenous LA, or lung gangrene.

## Case presentation

We present a case of a 68-year-old woman admitted to the Thoracic Surgery Department of University Hospital “St. Ivan Rilski”, Medical University of Sofia, Sofia, Bulgaria, with complaints of a dry cough, fever up to 39ºC, palpitations, fatigue, and diminished physical activity since two months. The patient had primary pneumonia two months ago and was treated by her general practitioner for seven days with amoxicillin/clavulanic acid 3x1 g, followed by a five-day course with clarithromycin sustained release (SR) 500 mg/d. After this suffering the temperature resolved, but the patient had a persistent dry cough. As a smoker, it was typical for her and she did not pay any attention. After this ambulatory therapy, a control x-ray was not performed. In these two months before hospitalization, her physical activity and well-being progressively worsened: she lost 6 kg and had night sweats and chills in addition to the cough. Three days before being admitted to the intensive care unit (ICU) she had a 39ºC temperature and was obnubilated and confused. Her x-ray upon admission was a round infiltrate, not well demarcated from the surrounding lung parenchyma. Since the patient had a history of pneumonia two months ago and none of her comorbidities was suggested as a risk factor for LA (arterial hypertension II grade, bilateral hip joint replacement - years ago), the initial working diagnosis of physicians was cavitary lung cancer.

During the first seven days of hospitalization, she was empirically treated with cefoperazone/sulbactam 2x2 g, levofloxacin 500 mg/d, metronidazole 3x500 mg/d, fluconazole 200 mg/d. The patient did not improve by the third day of the therapy. She kept febris continua (temperature at 38.8-38.9ºC) and had persistent non-productive dry cough. Hemocultures for aerobes, anaerobes, and fungi were collected, yielding a negative result. Fibrobronchoscopy was performed and did not detect any lesions past the subsegmental bronchi bilaterally. Bronchoalveolar lavage and histological specimens were obtained. The microbiological agents harvested from the lavage were *Achromobacter* and *Candida crusei*. Based on the antibiogram, the therapy was switched to amikacin 1 g/d, imipenem/cilastatin 4x500 g/d, and voriconazole (loading dose), followed by a maintenance dose of 200 mg/d for 11 days. The histological samples showed purulent inflammation with an absence of atypical cells, suggestive of cancer. The initial diagnosis of cavitary lung cancer was not verified and none of the above-mentioned combinations of wide-spectrum antibiotic agents proved to be effective. The patient additionally lost weight and developed anemia, hypoproteinemia, and dyselectrolytemia. The x-ray findings progressively worsened from a round infiltrate to a cavitary lesion with an air-liquid level. The aggressive antibacterial and antifungal therapy did not prevent the negative course of the disease, therefore the patient was offered for surgical treatment. The general examination of the patient upon admission to the thoracic surgery department showed slightly impaired general condition, cachexia, and sinus tachycardia at 110 bpm. The laboratory parameters were: WBC at 15.42x10^9/L, RBC at 3.29x10^12/L, hemoglobin (HGB) at 98 g/L, hematocrit (HCT) at 0.289 L/L, sedimentation rate at 140 mm/h (Table 1). All other findings were within the normal range.

**Table 1:** Laboratory findings and their normal range in the patient with gangrenous lung abscess. HGB: hemoglobin, HCT: hematocrit.

Parameter	Laboratory findings	Normal range
WBC	15.42x10^9/L	3.50-10.50x10^9/L
RBC	3.29x10^12/L	3.70-5.90x10^12/L
HGB	98 g/L	120-180 g/L
HCT	0.289 L/L	0.36-0.53 L/L
Sedimentation rate	140 mm/h	0-30 mm/h

**Figure 1:**
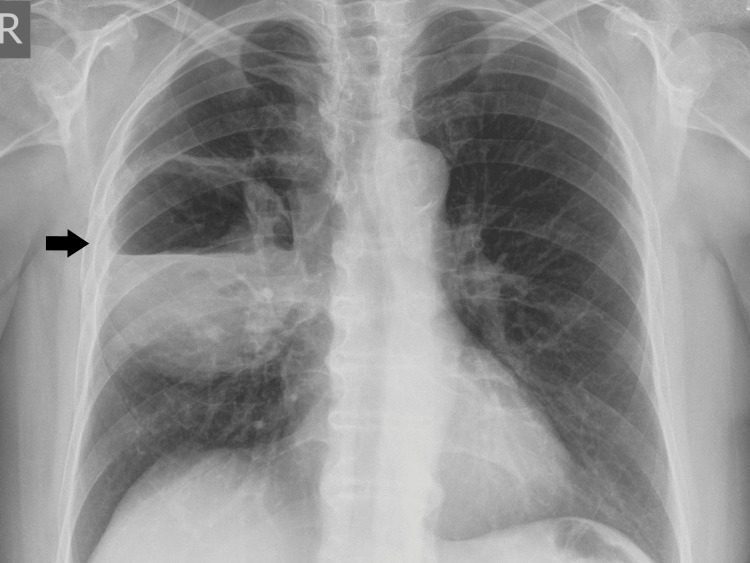
Chest x-ray, presenting an oval mass (arrow) in the right upper and middle lung lobes.

The chest x-ray showed a cavity with an unevenly thickened wall and air-fluid level in the upper and middle lobes of the right lung (Figure 1).

**Figure 2:**
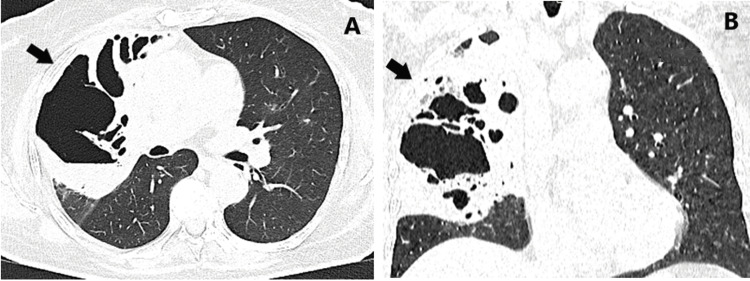
Chest CT in axial (A) and coronary (B) axis, presenting an abscess cavity (arrows) with destruction of the nearby parenchyma.

The computed tomography (CT) described a well-demarcated oval mass, that was with high colloidal density of liquid equivalent content filled with air bubbles. The lesion had a wall-thickness up to 3 mm, axial size was 78/72 mm, and coronary size 80 mm. A lot of perifocal smaller zones of consolidations might be observed cranial to the lesion in the same upper lobe (Figures 2A, 2B).

**Figure 3:**
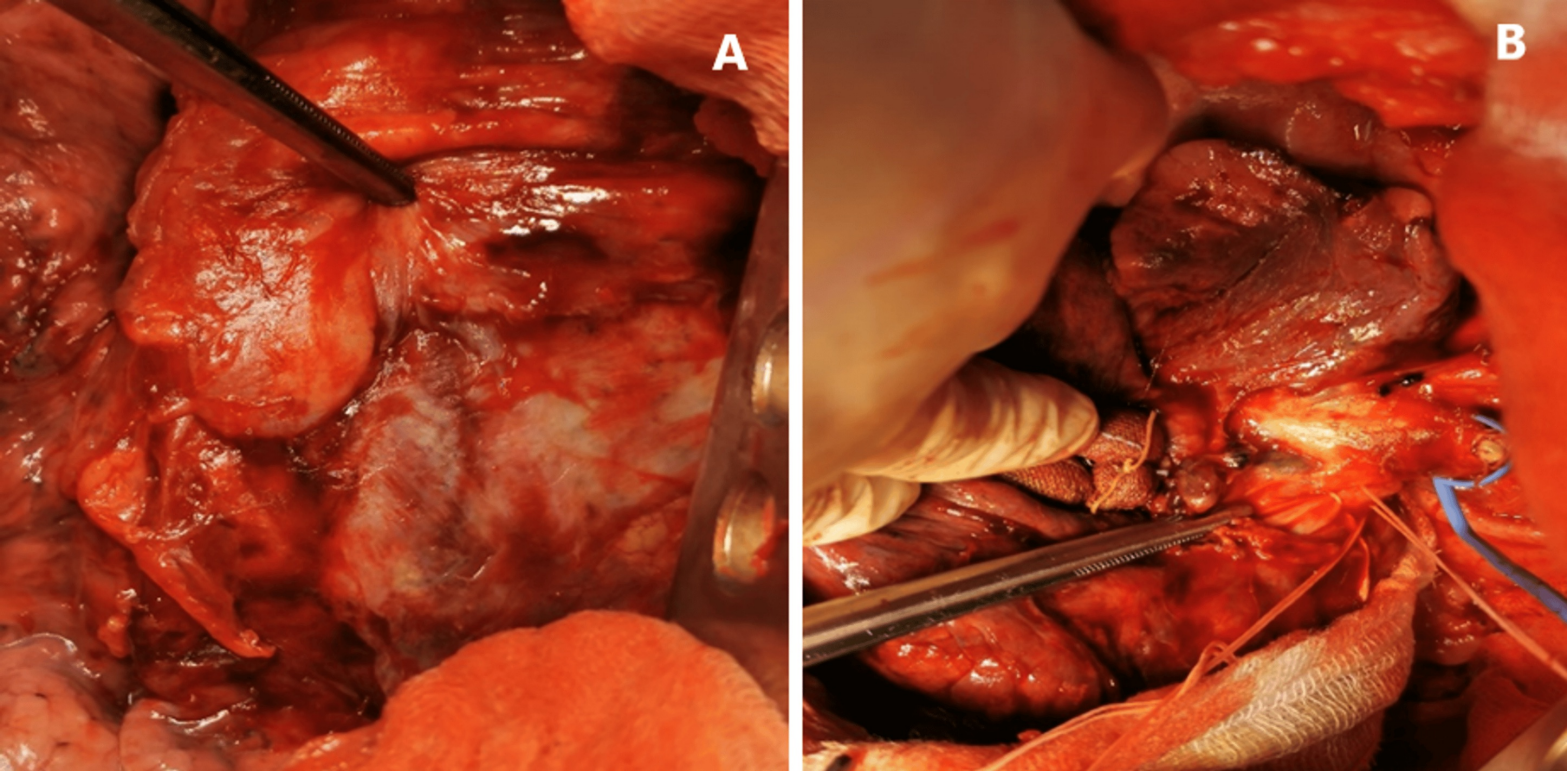
Intraoperative overview after the thoracotomy (A) and dissection of the hilar elements (B).

By means of right muscle-sparing dorsolateral thoracotomy, a moderately dense lesion with dimensions of 80/80 mm engaging the upper and middle lobes of the lung was palpated (Figures 3A, 3B). The interlobar sulcus was totally obliterated and inflammatory modified. Massive adhesions of the above-mentioned lesion with the parietal pleura were present, as in this place the mass was dissected through the extrapleural plane. A right upper bilobectomy was performed after separate manipulation of the hilar elements. The dissection of the latter was very difficult due to the inflammatory-fibrotic changes. Several hilar lymph nodes were intimately attached around the segmental arteries for the upper and middle lobes and were gently removed. A pleural catheter 24 Fr under active aspiration was inserted and thoracotomy was closed layer by layer.

**Figure 4:**
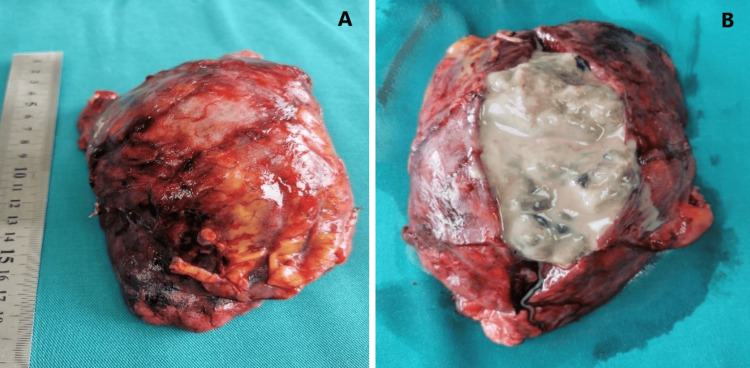
Macroscopic picture of the specimen (upper and middle lobes) (A) and after the section of its wall (B).

After the resection of the specimen, the macroscopic features of a gangrenous abscess engaging the upper and middle lobes were found (Figures 4A, 4B).

**Figure 5:**
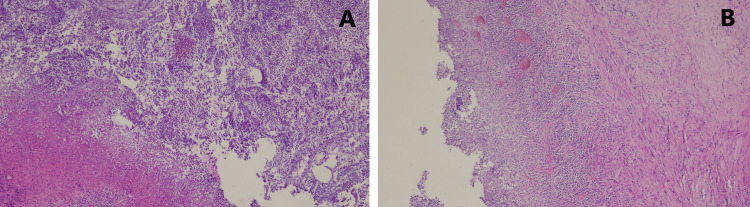
Histopathological findings in the patient with gangrenous lung abscess showing destruction of lung parenchyma from abscessing inflammatory process (A) and (B).

The histological result showed purulent bronchitis and bronchiolitis, lobular pneumonia, and an abscess cavity in the phase of organization. Foci of reactive squamous cell metaplasia in the lung parenchyma nearby, fibroblast plugs in the alveolar lumens (obliterating bronchiolitis), and reactive proliferation of pneumocytes and intra-alveolar accumulation of foamy macrophages were also described (Figure 5).

**Figure 6:**
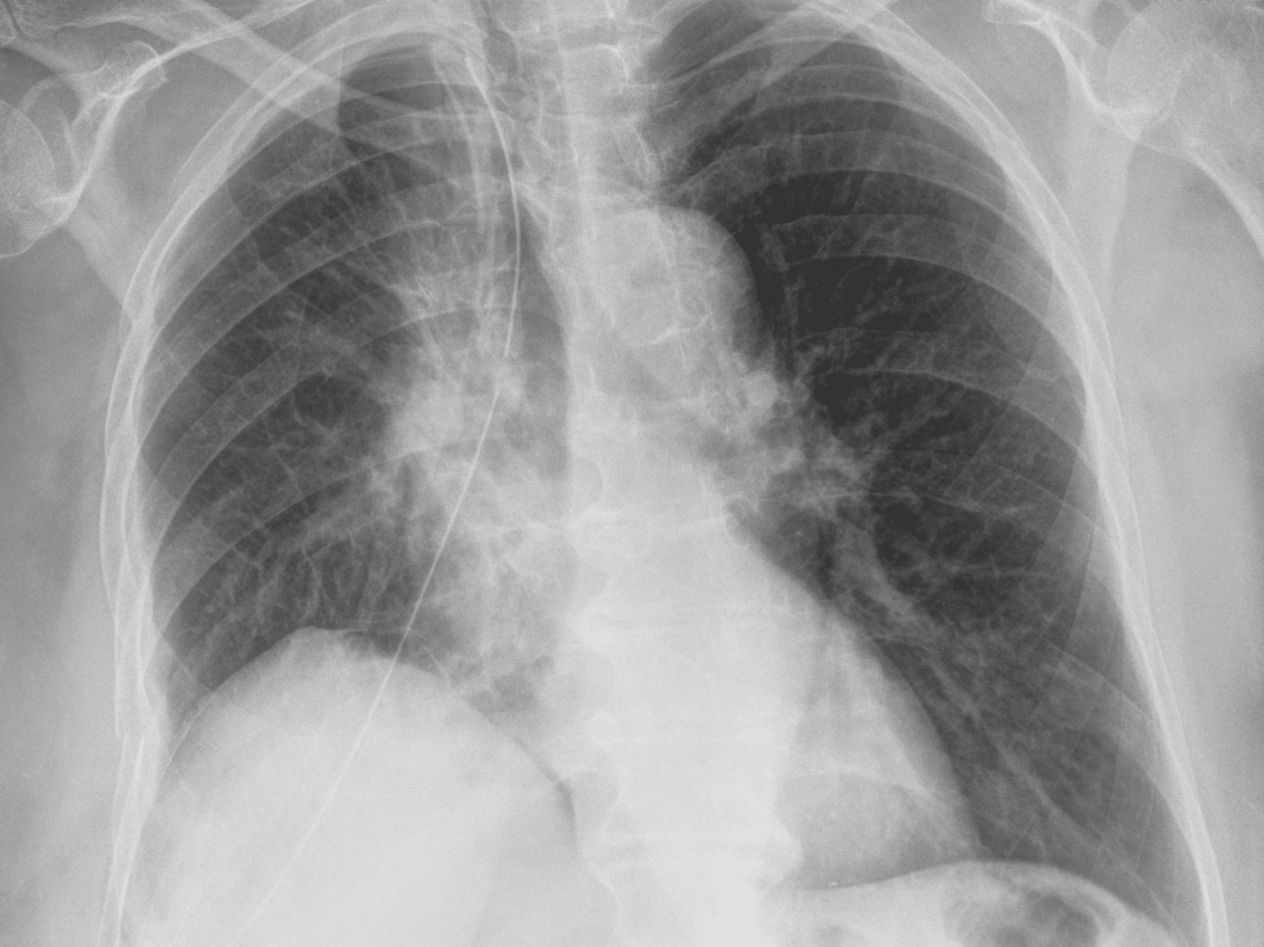
Chest x-ray on the 6th postoperative day just before removal of the thoracic catheter.

The postoperative period was uneventful with no complications. The chest x-ray on the 6th postoperative day before extraction of the drainage showed totally aerated lung parenchyma (Figure 6). The patient was discharged on the 7th day after surgery. 

## Discussion

LA and pleural empyema are among the most common complications of community-acquired pneumonia, especially in patients with comorbidities such as obesity and bronchial asthma [2]. The presence of LA and pleural empyema simultaneously occurs in some patients [3].

We present a 68-year-old woman in whom a right upper bilobectomy was performed due to a gangrenous abscess of the right lung. The clinical case is presented as a consequence of uncontrolled ambulatory treatment in a woman with apparently no obvious risk factors for necrotizing pneumonia and LA, which led to misdiagnosis and delayed adequate treatment. Before surgical resection, the patient was treated for three weeks in the ICU with a various combination of wide-spectrum antibiotics with a lack of effect, deterioration, and development of gangrenous LA. Moreover, we would like to emphasize the role of surgery as the utmost important treatment method in all patients with gangrenous LA with no alternative.

There are different classifications for LA. According to the duration of the LA, it could be acute (duration below six weeks) or chronic (duration above six weeks); according to the etiology, it could be primary (aspiration-associated, i.e., aspiration of oropharyngeal secretions, necrotizing pneumonia, immune-deficiency) or secondary (bronchial obstruction, hematogenic dissemination, direct engagement along a mediastinal infection, from the subphrenic space or from concomitant lung diseases). Depending on the type of distribution, the LA may be divided into bronchogenic (aspiration of oropharyngeal secretions, bronchial obstruction of tumor, foreign body, enlarged lymph nodes, congenital malformations) or hematogenic (sepsis, infectious endocarditis, septic thromboemboli) [4]. LA can also be divided into solitary or multiple, depending on the number of cavities; uni- or bilateral if one or both of the lungs are engaged, or acquired in the community or nosocomial.

In most cases, LA is caused by a couple of microorganisms, as the most common among them are aerobes such as *Klebsiella pneumoniae*, *Staphylococcus aureus*, *Streptococcus pneumoniae*, and *Pseudomonas aeruginosa* [5]. Anaerobic species like Bacteroides fragilis as well as uncommon species as *Mycobacterium* or *Aspergillus *may also rarely be isolated [4]. After a long antibiotic course, the samples may be sterile. The microbiological diagnosis may be verified by inoculation of samples, taken from expectoration of sputum, broncho-alveolar lavage, transtracheal aspiration, thoracentesis of pleural effusion or hemoculture when sepsis is present. In the clinical case we demonstrate, the microbiological agents, isolated after bronchial aspiration were *Achromobacter* and *Candida crusei*, which are not typically considered in LA pathogenesis. The microbiology of the lavage did not facilitate the proper diagnosis of necrotizing pneumonia and deterred the appropriate therapeutic algorithm. Moreover, the cultivation of microorganisms was not prevented from a recent antibiotic therapy, as the patient had been taking it more than two months ago. A control x-ray after this primary ambulatory treatment was not performed, so we had no reason to think of persistent inflammatory foci. In this therapeutic gap of two months, the patient had gone through a tooth extraction, which had been followed by the intake of amoxicillin/clavulanic acid 2x1 g for a week. We can only speculate for a transient bacteremia after the procedure, or it might be, that, this short antibiotic course suppressed and delayed the natural evolution of the primary lung inflammation.

The clinical presentation in the gangrenous abscess is most commonly severe; septic temperature over 38ºC, cough, dyspnea, thoracic pain, weight loss, loss of appetite, night sweating, and anemia [6]. Sometimes hemoptoe may also be present. In this clinical case, the major complaints were uncommon and included dry cough, temperature up to 39ºC, tachycardia, fatigue, and diminished physical activity lasting for more than two weeks. These were followed by an abrupt clinical deterioration; febris continua, syncope, obnibulation, and anemia. This subtle clinical course may be due to persistent, not fully eradicated bacterial inflammation in a person with generally preserved and uncompromised immune status.

LA is most commonly seen in men, alcohol abusers, patients above 50 years of age, in cases with loss of consciousness, as it is most commonly observed after aspiration [4]. Other risk factors are diabetes, mechanical ventilation, immune suppression, etc. [7]. Our patient had no predisposing risk factors which may have deterred the diagnosis and obscured the natural evolution of the disease.

In LA, associated with aspiration pneumonia, the apical segments of the lower lung lobes or the dorsal segments of the upper lobes are most often engaged, as the right side is more commonly affected in comparison to the left one [8]. If hematogenic dissemination is observed there is no predominant location of the process. In our case, the abscess was in the right lung, the right upper and middle lung lobes.

The chest x-ray presented an oval lesion with well-demarcated boundaries. On CT, LA is detected as a liquid containing cavitary lesion, that is with thick walls, sometimes with an air-fluid level, uneven luminal surface, and surrounding consolidation [9].

The differential diagnosis is complex and complicated. A cavitary lung cancer, tuberculosis, encapsulated empyema, infected bulla, hiatal hernia, pulmonary hematoma, hydatid cysts, cavitary lung infarction, foreign body aspiration, or septic pulmonary emboli should be considered [6].

The treatment of LA in most cases begins conservatively with antibiotic courses, depending on the etiological agents. However, it is not always successful, especially if some risk factors like a huge cavity>6 cm in diameter, advanced age, immunosuppression or cancer are present [1]. If life-threatening hemoptysis, broncho-pleural fistula, or rupture of the abscess in the pleural cavity occurs, surgical treatment is the first method of choice. Surgical resection should be strongly indicated when a patient's condition deteriorates despite aggressive medical treatment and when a gangrenous LA is proved on CT. The endoscopic drainage method is a modern minimally invasive method with increasing success. Placement of a percutaneous tube drain under ultrasound or CT guidance is also the method of choice. It is better than the conservative approach in regards to the total effectiveness and also reduces the length of hospital stay and the number of days with fever [10]. CT-guided percutaneous drainage is a safe method in patients with a lack of response to antibiotic treatment and is an alternative to open lung surgery [11]. The open- or video-assisted resection of the lung by atypical resection, segmentectomy, lobectomy, or pneumonectomy, and decortication are the means of choice when other treatment methods are ineffective. Lung resection is indicated in the case of failure of the percutaneous drainage or when necrosis distributes to the other lobes [12]. In gangrenous abscesses, especially the gigantic ones (diameter larger than 7 cm), that engage two or three lobes of the lung, an open surgery (thoracotomy) is usually demanded. Open surgery for removal of the part or whole lung should be done in pulmonary gangrene, as it is impossible to drain the infected lung parenchyma [13]. Chance for antibiotic effectiveness and healing are given as follows: in lung gangrene up to one to two weeks, gangrenous LA two to four weeks, and in LA six weeks. When there is progression of the disease and deterioration in the general state, resection is performed. Surgery is commonly performed after conservative management for two to four weeks from the onset of the disease, which contributes to the development of firm adhesions in the chest cavity and pulmonary hilum.

In the presented case here we performed directly open surgery because of the size of the cavitary mass, the thick walls, the encompassment of two lobes, and the presence of huge inflammatory changes in the surrounding parenchyma. The operative intervention was a complex one due to the inflammatory change of the hilar structures of the right lung. Despite the total absence of the interlobar sulcus due to inflammatory obliteration, we were able to preserve the lower lung lobe. During surgery, care must be taken to avoid spilling contents from the abscess cavity into the contralateral lung and contaminating the latter. The surgical treatment of gangrenous abscesses is difficult and challenging due to the heavy inflammation of both pulmonary parenchyma, blood vessels, and bronchi. This makes their dissection and processing extremely difficult and risky with a high probability of iatrogenic injuries. Avoiding pneumonectomy in such cases with an infected space is recommended, as postsurgical complications after it is at a high rate and with disastrous consequences.

The complications after surgical treatment are insufficiency of the bronchial stump and development of pyopneumothorax, pleural empyema, hemorrhage, hematogenic dissemination, sepsis, and polyorgan failure. In our clinical case, no complications occurred. Predictors for a fatal outcome are sepsis, septic complications (pulmonary leakage, pleural empyema), septic multiorgan failure (acute respiratory or kidney failure), and accompanying comorbidity (Charlson comorbidity index >30), with the volume of surgical resection having no significant impact on mortality [14].

## Conclusions

We present a clinical case of gangrenous LA in a woman who had been ambulatory treated for pneumonia and had no risk factors for necrotic inflammation. We highlight the importance of controlled antibiotic therapy (both laboratory and x-ray), since the inappropriate and ineffective antimicrobial courses in subjects with preserved immune status may obscure the typical clinical presentation of necrotizing inflammation. The indolent pathogenesis, the atypical symptoms, and the delayed therapeutic approach in our patient ended in gangrenous LA. Due to misdiagnosis and adequate therapy, the natural evolution of anaerobic inflammation and necrosis was irreversible and could not be controlled under standard treatment. Living in the era of growing antibiotic resistance, cases like this may become more common and should be kept in mind. Surgical resection should be strongly indicated when a patient's condition deteriorates despite aggressive medical treatment and when a gangrenous LA is proved on CT. Moreover, although the performance of surgical treatment is the only method of choice, it is often very difficult, time-consuming, and complicated as both necrotic and fibrotic abnormalities deter the procedure and prolong the recovery after it. The gangrenous LA is very challenging for resection because of the inflammatory abnormalities in the lung hilum that obscure the normal anatomical planes. Surgical intervention is the life-saving method of choice in such cases and offers the greatest chance of definitive treatment. Thus, timely diagnosis and controlled antibiotic treatment decrease the risk of therapeutic failure, complications, and mortality.
